# The impact of metabolic syndrome on regional ventilation and perfusion in ARDS: an observational cohort study using electrical impedance tomography

**DOI:** 10.1186/s40635-026-00883-8

**Published:** 2026-03-11

**Authors:** Timothy G. Gaulton, Marcus Victor, Glasiele Alcala, Roberta Ribeiro De Santis Santiago, Florencia Rodriguez Sendic, Yi Xin, Stefano Spina, Cristina Mietto, Lorenzo Berra, Maurizio Cereda

**Affiliations:** 1 Department of Anesthesiology, Massachusetts General Brigham, Boston, MA USA; 2 Harvard Medical School, Boston, MA USA; 3 Respiratory Care Department, Massachusetts General Hospital, Boston, MA USA

**Keywords:** Metabolic syndrome, Regional ventilation and perfusion, Acute respiratory distress syndrome, Electrical impedance tomography

## Abstract

**Background:**

Acute Respiratory Distress Syndrome (ARDS) is characterized by severe hypoxemia from heterogeneous impairments in regional ventilation and perfusion. Metabolic syndrome, a combination of central obesity, insulin resistance, hypertension, and dyslipidemia, affects over one-third of adults worldwide and is associated with systemic inflammation, endothelial dysfunction, and increased ARDS risk. Whether metabolic syndrome alters regional ventilation and perfusion distributions in ARDS remains unknown.

**Methods:**

We performed a retrospective cohort study of 25 mechanically ventilated patients with ARDS evaluated by the Massachusetts General Hospital Lung Rescue Team from 2020 to 2025. After a recruitment maneuver and decremental PEEP titration in the supine position, we quantified regional ventilation and perfusion distributions using electrical impedance tomography across a 32 × 32 pixel impedance matrix. We used Bayesian regression modeling adjusted for age, severity of illness, and ARDS etiology to define associations between metabolic syndrome and regional distributions.

**Results:**

25 patients were included, of whom 44% (n = 11) had metabolic syndrome. The mean age was 52 (16) years, PaO₂/FiO₂ was 151 (61), and 48% (n = 12) had pulmonary ARDS. Patients with metabolic syndrome were younger and had higher respiratory system compliance. In dorsal lung regions, metabolic syndrome was associated with reduced ventilation distribution (adjusted mean difference: − 5.84%, 95% credible interval: − 12.29 to 1.17, posterior probability of decrease = 95.2%) and modest, uncertain reductions in perfusion distribution (− 2.01%, 95% credible interval: − 9.25 to 5.41). The proportion of pixels with a low ventilation-to-perfusion impedance ratio showed a trend toward increase in dorsal lung regions in patients with metabolic syndrome (4.25%, 95% credible interval: − 4.49 to 13.1, posterior probability = 80.3%). Body mass index accounted for 40% of the difference in dorsal ventilation. The association between regional ventilation and perfusion distributions was similar between groups (interaction coefficient: 0.01, 95% credible interval: − 0.34 to 0.36).

**Conclusions:**

In ARDS, metabolic syndrome may be associated with reduced ventilation distribution to dorsal lung regions with smaller changes in perfusion distribution. The association between regional ventilation and perfusion distributions did not differ by metabolic syndrome. These exploratory findings suggest metabolic syndrome may primarily affect ventilation distribution rather than perfusion distribution in ARDS.

**Supplementary Information:**

The online version contains supplementary material available at 10.1186/s40635-026-00883-8.

## Introduction

A hallmark of Acute Respiratory Distress Syndrome (ARDS) is hypoxemia due to heterogeneous impairments of regional pulmonary ventilation and perfusion [1]. The spatial relationship between ventilation and perfusion not only determines hypoxemia severity and disease progression but likely modifies responses to mechanical ventilation. Given the persistently high incidence and mortality of ARDS [2] and the increasing evidence of ARDS as a heterogeneous syndrome [3], understanding how patient-level characteristics influence regional ventilation and perfusion using imaging is essential to improve disease phenotyping and refine treatment strategies.

Metabolic syndrome, defined by central obesity, insulin resistance, hypertension, and dyslipidemia [4], affects over one-third of adults worldwide and is associated with increased ARDS risk and severity [5]. The reasons for the increased vulnerability remain poorly defined, and existing management of ARDS does not consider metabolic characteristics. Whether metabolic syndrome alters regional lung physiology with implications for individualized treatment has not been established.

The mechanisms by which metabolic syndrome might alter regional lung physiology are multifactorial. While excess adiposity is known to compress the lung, [6] metabolic syndrome encompasses other systemic abnormalities including endothelial dysfunction, chronic inflammation, and altered lipid metabolism [7, 8]. These may increase pulmonary capillary permeability and edema formation[9], adding to lung weight and augmenting dorsal lung collapse beyond the effects of adiposity alone. Consistent with this mechanism, biomarker studies suggest that patients with metabolic syndrome exhibit more pronounced pulmonary endothelial injury during ARDS [10, 11]. Moreover, preclinical models of metabolic syndrome have demonstrated impaired pulmonary arterial vasoreactivity [7]. Ongoing perfusion to collapsed lung regions would increase physiologic ventilation to perfusion mismatch, potentially explaining the increased risk of ARDS and severity observed in metabolic syndrome.

Electrical Impedance Tomography (EIT) enables real-time, radiation-free measurement of regional ventilation and perfusion at the bedside [12, 13]. Unlike other respiratory monitors, EIT provides topographic mapping of ventilation and perfusion, making it ideally suited to investigate regional pulmonary physiology. We have previously validated regional perfusion changes measured by EIT against gold-standard imaging in ARDS [14]. At Massachusetts General Hospital, EIT is applied routinely to characterize regional heterogeneity and guide ventilatory management in patients with severe hypoxemia [15].

We conducted a retrospective cohort study of patients with ARDS who underwent EIT assessment to determine whether metabolic syndrome produces distinct ventilation-perfusion distributions. We tested three sequential hypotheses: (1) metabolic syndrome reduces ventilation to dorsal lung regions, (2) metabolic syndrome attenuates perfusion redistribution from dorsal regions, and (3) these combined responses alter the distribution of ventilation to perfusion (V/Q) impedance ratios.

## Methods

### Study design

We conducted a retrospective observational cohort study of patients with ARDS admitted to Massachusetts General Hospital. We followed the Strengthening the Reporting of Observational Studies in Epidemiology guidelines for our study design, analysis, and reporting (Supplementary Material 1) [16]. The Massachusetts General Hospital Institutional Review Board approved primary data collection (#2020P003196) and retrospective analysis and publication (#2024P003186).

### Patient selection

The study cohort consisted of all patients on invasive mechanical ventilation who underwent Lung Rescue Team assessment with EIT measurements of regional ventilation and perfusion between March 2020 and April 2025. The Lung Rescue Team is a multidisciplinary group that evaluates patients with acute hypoxemic respiratory failure using advanced physiologic monitoring [15].

### Metabolic syndrome definition

We defined metabolic syndrome based on National Heart, Lung, and Blood Institute criteria. Patients met criteria for metabolic syndrome if they had at least three of the following five components defined from electronic medical record review [4]: (1) central obesity, defined as body mass index ≥ 35 kg/m^2^ at hospital admission, (2) diagnosis of hypertension, (3) diagnosis of diabetes mellitus, (4) elevated triglycerides (≥ 150 mg/dL), (5) reduced HDL cholesterol (< 40 mg/dL in men, < 50 mg/dL in women). Direct laboratory values for triglycerides or HDL cholesterol were obtained from outpatient records within six months of admission, not including the index admission to avoid confounding of triglyceride levels by sedative use. Triglyceride values were available for 23 patients (92%) and HDL cholesterol for 22 patients (88%). For patients with missing laboratory values, these components were classified based on documented use of triglyceride or lipid-lowering medications prior to admission. We used BMI (≥ 35 kg/m^2^) as a surrogate for central obesity, as waist circumference is not routinely measured in our practice and has high correlation with BMI at this threshold [17].

### Covariates

We collected demographic variables (age, sex, race, Hispanic ethnicity), respiratory mechanics and gas exchange prior to the Lung Rescue Team assessment (PaO₂/FiO₂, respiratory system compliance, driving pressure), ARDS etiology (pulmonary [pneumonia, aspiration] versus extrapulmonary [sepsis, trauma]), Acute Physiology and Chronic Health Evaluation (APACHE II) score, vasopressor use, and time from ARDS onset to assessment.

### Lung rescue team and electrical impedance tomography assessment

EIT assessment used a commercial device (Enlight 2100, Timpel Medical, São Paulo, Brazil) with a circumferential 32-electrode belt positioned at the fourth intercostal space [18]. Electrodes deliver alternating electrical currents (~ 10 mA) at 50 Hz. Voltage changes are then reconstructed into 32 × 32 pixel impedance maps.

After connecting patients to EIT, the Lung Rescue Team performed a recruitment maneuver followed by a decremental positive end-expiratory pressure (PEEP) trial in 2 cmH_2_0 steps in the supine position [19]. Plateau pressures during the recruitment maneuver were individualized to patient body habitus and hemodynamic tolerance, with higher pressures used in patients with severe obesity to overcome elevated pleural pressures, consistent with published protocols for this population [19, 20]. Real-time EIT monitoring provided continuous assessment of regional overdistension during the maneuver. At each decremental PEEP step, EIT calculates the percentage of regional overdistention and collapse based on pixel level changes in impedance. Following recruitment and the decremental PEEP trial, the Lung Rescue Team selected optimal PEEP as the point that minimized regional overdistention and collapse [18]. Neuromuscular blockade and sedation were maintained by the clinical team.

All EIT assessments, including perfusion measurements, were performed as part of routine clinical care by the Lung Rescue Team and were not research-specific procedures. The saline bolus perfusion technique is performed routinely at our institution for patients with severe hypoxemia, consistent with published clinical protocols [12, 21]. The bolus of hypertonic saline follows established methodology for EIT-based perfusion measurement [22] and represents a negligible osmolar and volume load relative to doses used in clinical resuscitation. All patients were continuously monitored with invasive arterial blood pressure and pulse oximetry during Lung Rescue Team assessments. No adverse events attributable to the hypertonic saline bolus have been observed.

Regional ventilation and perfusion measurements used for the present analysis were obtained at a single timepoint, after the recruitment maneuver and decremental PEEP titration were completed and the EIT-determined optimal PEEP was set. Regional ventilation was quantified from impedance changes during tidal breathing, averaged over 10 breaths, using inspiratory and expiratory flow signals from a proximal sensor. Regional perfusion was measured using the indicator dilution method [23]. Following an end-expiratory breath hold, 10 mL of 7.5% hypertonic saline was rapidly injected via a central venous catheter. High-frequency and cardiac-related impedance changes were filtered based on frequency analysis. Pixels with amplitude < 10% of maximum were excluded. Impedance-time curves for each pixel were fitted to a gamma function, and the maximum slope was used to generate a regional perfusion map. Ventilation and perfusion impedance signals were normalized to range from 0 to 1 across the whole lung map. Ventral and dorsal regions of interest were defined by dividing the 32 × 32 pixel impedance matrix into two equal halves along the ventral-dorsal axis at the midpoint. This geometric division is based on the pixel matrix and does not incorporate anatomical correction, consistent with established EIT methodology [24].

### Outcomes

Our primary outcomes were the distribution of ventilation and perfusion to dorsal lung regions, defined as the percentage of either perfusion or ventilation in that lung region compared to the total map. Secondary outcomes included the topographic distribution of V/Q impedance ratios. For each region, we calculated the proportion of pixels with low (< 0.5) or high (> 2.0) V/Q impedance ratios based on predetermined thresholds [25]. Dorsal-to-ventral ratios were calculated by dividing the dorsal percentage by the ventral percentage (where ventral = 100%—dorsal). A ratio of 1.0 indicates equal distribution between regions. Values greater than 1.0 indicate dorsal predominance, while values less than 1.0 indicate ventral predominance.

### Statistical analysis

#### Descriptive statistics

We summarized baseline characteristics overall and by metabolic syndrome as mean (SD) or median [IQR] depending on their distribution. We calculated standardized mean differences (SMD) to assess covariate balance.

#### Bayesian regression models

We used Bayesian inference because it accommodates bounded outcome data, allows incorporation of prior physiological knowledge about ventilation and perfusion distributions, and appropriately quantifies uncertainty when sample sizes are modest [26]. We performed adjusted analyses using Bayesian regression implemented with the brms package (R version 4.5) [27]. Based on distribution diagnostics, we modeled ventilation and perfusion distribution using Gaussian likelihoods and V/Q impedance ratios using Student-t likelihoods to accommodate skewness and outliers. We used weakly informative priors based on physiological constraints and prior EIT literature (Supplementary Material 2), following recommendations for Bayesian analysis in critical care medicine [25, 28, 29]. Models adjusted for age, APACHE II score, and ARDS etiology [2]. We did not adjust for baseline respiratory mechanics, oxygenation, or PEEP level as these variables lie in the causal pathway between metabolic syndrome and ventilation-perfusion impairment. Four Markov chains with 4,000 iterations each (1,000 warmup) were run. Model convergence was assessed using R-hat statistics (< 1.01) and effective sample sizes (ESS > 400). Model fit was evaluated using leave-one-out cross-validation with Pareto-k diagnostics to identify influential observations. All Bayesian models converged successfully (R-hat < 1.01, ESS > 400) with no highly influential observations (max Pareto-k < 0.7). Posterior predictive checks confirmed adequate model fit that adequately captured observed data distributions (Supplementary Material 3).

#### Regional ventilation and perfusion associations

To test whether metabolic syndrome modified the association between regional ventilation and perfusion distributions, we fit a Bayesian interaction model predicting dorsal perfusion distribution from dorsal ventilation distribution, metabolic syndrome status, and their interaction. A significant interaction would indicate that the association between regional ventilation and perfusion distributions differed by metabolic syndrome.

#### Pathway decomposition

We decomposed the association between metabolic syndrome and V/Q impedance ratios into direct and indirect pathways by calculating the product of: (1) the association of metabolic syndrome with ventilation or perfusion distribution, and (2) the association of ventilation or perfusion distribution with V/Q impedance ratios in dorsal lung regions.

#### Sensitivity analyses

We repeated our primary analyses with adjustment for sex. We also repeated the primary model of ventilation distribution with additional adjustment for BMI to determine whether the association between metabolic syndrome and ventilation distribution was mediated by BMI alone versus the other components of metabolic syndrome.

#### Reporting

We reported posterior mean differences with 95% credible intervals (CrI) and posterior probabilities. Credible intervals represent the range within which the true effect lies with 95% probability and posterior probabilities quantify the certainty that an effect is in a particular direction. Posterior probabilities ≥ 95% indicate credible evidence of an effect in that direction. All analyses were performed using R version 4.5.2 (R Core Team, 2025).

#### Sample size estimation

This was an exploratory analysis of a clinical cohort with sample size determined by availability of patients who underwent comprehensive EIT assessment during the study period. No formal sample size calculation was performed.

## Results

### Patient characteristics

Of 33 patients assessed with EIT, 7 were excluded due to not meeting criteria for ARDS (unilateral disease or PaO₂/FiO₂ > 300) and 1 patient was excluded for having an EIT assessment > 7 days from ARDS onset. Our study cohort included 25 patients with ARDS, of whom 44% (n = 11) had metabolic syndrome. The mean PaO₂/FiO₂ was 151 (SD 61) and 48% (n = 12) had a pulmonary etiology of ARDS. Characteristics are presented in Tables 1 and 2. Patients with metabolic syndrome were younger (mean age 45 vs 57 years, SMD = 0.82) and had similar etiologies of ARDS and illness severity compared to patients without metabolic syndrome. Prior to recruitment and PEEP titration, patients with metabolic syndrome had higher respiratory system compliance (36 vs 27 mL/cmH₂O, SMD = 0.71) and lower PaO₂/FiO₂ (138 vs 161, SMD 0.37. Following recruitment and decremental PEEP titration, EIT-selected PEEP was higher in patients with metabolic syndrome (14.9 vs 11.1 cmH₂O, SMD = 0.85), with higher post-titration respiratory system compliance (47.4 vs 33 mL/cmH₂O, SMD = 0.89) and lower residual EIT derived collapse (3% vs 6%, SMD = 0.89). Time from ARDS onset to EIT assessment was the same in each group.

**Table 1:** Characteristics of 25 patients with ARDS

Characteristic	Overall (N = 25)	Metabolic Syndrome		SMD
		No (N = 14)	Yes (N = 11)	
Age (years)	51.8 (16.3)	57.4 (13.9)	44.8 (16.9)	0.82
*Sex*				
Female	9 (36%)	6 (43%)	3 (27%)	0.33
Male	16 (64%)	8 (57%)	8 (73%)	
*Race*				
White	23 (92%)	12 (86%)	11 (100%)	0.58
Black	1 (4%)	1 (7%)	0 (0%)	
Asian	1 (4%)	1 (7%)	0 (0%)	
Hispanic Ethnicity	4 (16%)	2 (14%)	2 (18%)	0.11
Body Mass Index (kg/m^2^)	37.2 (13.3)	28.7 (7.4)	48.0 (11.2)	2.08
Triglycerides (mg/dL)	158 (88.9)	102 (39.7)	224 (86.5)	1.86
HDL Cholesterol (mg/dL)	41.0 (16.1)	46.5 (15.8)	32.6 (13.3)	0.93
Hypertension	16 (64%)	8 (57%)	8 (73%)	0.33
Diabetes Mellitus	9 (36%)	4 (29%)	5 (45%)	0.36
*ARDS Etiology*				
Extrapulmonary	13 (52%)	8 (57%)	5 (45%)	0.24
Pulmonary	12 (48%)	6 (43%)	6 (55%)	
APACHE II Score	20 (8.7)	22 (8.4)	18 (8.7)	0.51
Vasopressor use	18 (72%)	12 (86%)	6 (55%)	0.72
Time from ARDS Onset to Assessment (days)	1.0 [1.0, 2.0]	1.5 [0.2, 2.8]	1.0 [1.0, 2.0]	0.01

Data are presented as mean (SD), median [IQR], or n (%). SMD = standardized mean difference; ARDS = acute respiratory distress syndrome; APACHE II = Acute Physiology and Chronic Health Evaluation; HDL = high-density lipoprotein; BMI = body mass index

**Table 2:** Ventilatory and EIT parameters

Parameter	Overall (N = 25)	Metabolic syndrome		SMD
		No (N = 14)	Yes (N = 11)	
**Pre PEEP titration**				
PEEP (cmH₂O)	11.6 (5)	10.4 (4.9)	13.2 (5)	0.56
PaO₂/FiO₂	151 (61.2)	161 (66.4)	138 (54.1)	0.37
Driving Pressure (cmH₂O)	13.3 (3.0)	14.3 (3.1)	12.0 (2.5)	0.82
Respiratory System Compliance (mL/cmH₂O)	30.9 (12.4)	27.2 (10.8)	35.6 (13.1)	0.71
**Post PEEP titration**				
EIT-selected PEEP (cmH₂O)	12.8 (4.8)	11.1 (4.5)	14.9 (4.5)	0.85
Respiratory System Compliance (mL/cmH₂O)	39.4 (17.4)	33 (15.9)	47.4 (16.4)	0.89
Residual collapse at EIT PEEP (%)	4.6 (3.7)	6 (4.3)	3 (2.1)	0.89
Residual overdistension at EIT PEEP (%)	5.9 (3.8)	6.5 (4.9)	5.2 (2.2)	0.36

Values are mean (SD). SMD = standardized mean difference

PEEP, positive end-expiratory pressure; EIT, electrical impedance tomography; PaO₂/FiO₂, ratio of arterial partial pressure of oxygen to fraction of inspired oxygen

Collapse and overdistension data from EIT were available for 21 patients (11 without metabolic syndrome, 10 with metabolic syndrome)

### Association of metabolic syndrome to regional ventilation and perfusion distribution

**Fig. 1:**
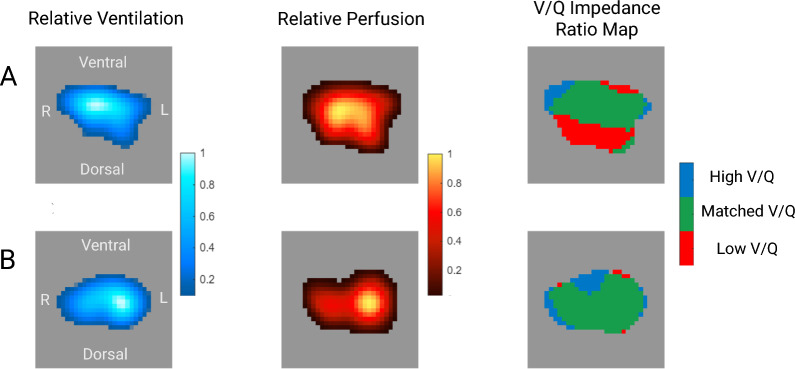
Electrical impedance tomography maps of regional ventilation and perfusion in two patients with ARDS. Panel **A**: patient without metabolic syndrome. Panel **B**: patient with metabolic syndrome. For each patient, the left column displays regional ventilation maps derived from tidal impedance changes (blue-white scale), the middle column displays relative perfusion maps derived from the indicator bolus technique (red-yellow scale), and the right column displays V/Q impedance ratio maps. Red represents low V/Q impedance ratios (< 0.5), blue represents high V/Q impedance ratios (> 2), and green represents matched V/Q impedance ratios (0.5–2). Each patient map is normalized to its maximum signal intensity. Images are oriented with ventral at top; right lung is displayed on the left side of each image

**Fig. 2:**
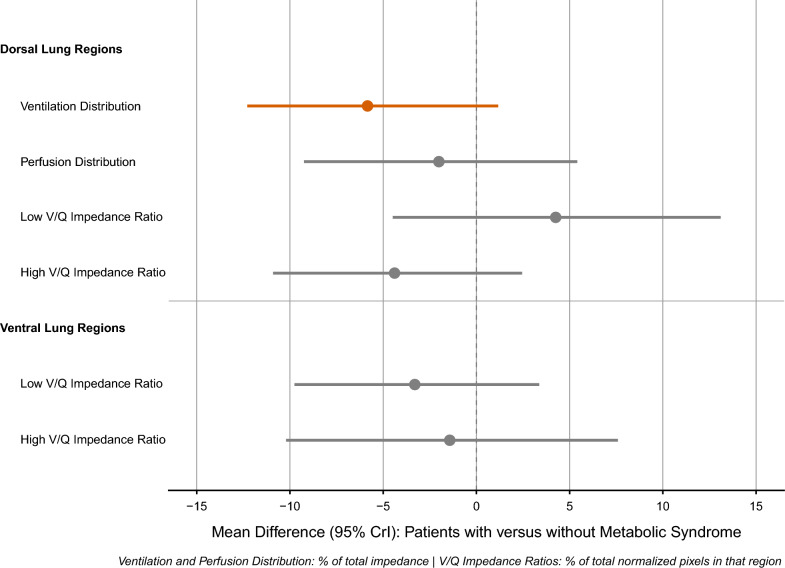
Effect of Metabolic Syndrome on Regional Lung Ventilation and Perfusion Distributions. Forest plot showing the association between metabolic syndrome and the distributions of ventilation, perfusion, and ventilation to perfusion impedance signals in dorsal and ventral lung regions. Points represent posterior mean differences (estimates in patients with metabolic syndrome minus patients without metabolic syndrome); error bars represent 95% credible intervals. A red line indicates posterior probability ≥ 95%

Topographic maps of regional ventilation and perfusion distribution from two patients in our cohort are shown in Fig. 1. Unadjusted ventilation distribution to dorsal lung regions was 38.6% (SD 14.8) in patients with metabolic syndrome versus 45.8% (SD 15.2) in those without (Supplementary Material 4). Unadjusted perfusion distribution to dorsal lung regions was 51.2% (SD 19.3) versus 54.7% (SD 18.1). After adjustment for age, APACHE II score, and ARDS etiology, metabolic syndrome was associated with a reduction in ventilation distribution to dorsal lung regions (mean difference: -5.84%, 95% CrI: − 12.29 to 1.17, posterior probability of decrease = 95.2%). Perfusion distribution showed a lower reduction with more uncertainty (mean difference: − 2.01%, 95% CrI: − 9.25 to 5.41, posterior probability of decrease = 68.8%). Adjusted results by metabolic syndrome are presented in Table 3 and Fig. 2.

**Table 3:** Regional Lung Ventilation and Perfusion by Metabolic Syndrome

Outcome	Metabolic Syndrome*		Difference (Yes – No)	Pr(direction)
	No (N = 14)	Yes (N = 11)		
Dorsal Lung Regions**				
Ventilation Distribution	45.8 [23.8, 67.3]	40.1 [18.4, 62.0]	− 5.8 [− 12.3, 1.2]	95.2% ↓
Perfusion Distribution	54.7 [26.4, 82.1]	53.1 [25.8, 81.2]	− 2.0 [− 9.2, 5.4]	68.8% ↓
Low V/Q Impedance Ratio	22.9 [− 36.6, 80.4]	27.7 [− 28.9, 87.1]	4.25 [− 4.49, 13.1]	80.3% ↑
High V/Q Impedance Ratio	14.4 [− 11.4, 39.5]	11.0 [− 14.2, 36.1]	− 3.4 [− 9.9, 3.3]	85.9% ↓
**Ventral Lung Regions**				
Low V/Q Impedance Ratio	10.6 [− 14.5, 37.0]	5.9 [− 19.2, 30.8]	− 4.4 [− 11.0, 2.3]	90.3% ↓
High V/Q Impedance Ratio	30.7 [− 24.2, 84.4]	29.4 [− 25.3, 84.3]	− 1.4 [− 10.4, 7.8]	58.8% ↓

^*^Values are predicted means [95% credible intervals] from Bayesian regression models

adjusted for age, APACHE II score, and ARDS etiology. Predictions calculated at mean age (52 years), mean APACHE II score (20), and extrapulmonary ARDS etiology. Pr(direction) = posterior probability of the specified direction (↓ decrease, ↑ increase). Probabilities ≥ 95% indicate credible evidence of an association

^**^Distribution—% of total impedance; V/Q (ventilation to perfusion)—% of normalized pixels in that region

Regional distribution analysis suggested that metabolic syndrome was associated with a ventral shift in ventilation distribution, with a lower dorsal-to-ventral ventilation ratio (mean difference: -0.34, 95% CrI: − 0.67 to 0.02, posterior probability of decrease = 96.8%). The perfusion ratio showed a similar directional trend (mean difference: − 0.15, 95% CrI: − 0.76 to 0.46).

In dorsal lung regions (Table 3), low V/Q impedance ratios trended higher in patients with metabolic syndrome (mean difference: 4.25%, 95% CrI: − 4.49 to 13.1, posterior probability of increase = 80.3%) and high V/Q impedance ratios trended lower (mean difference: − 3.4%, 95% CrI: − 9.9 to 3.3, posterior probability of decrease = 85.9%) but this did not reach thresholds for a meaningful difference (95% posterior probability). In ventral lung regions, low V/Q impedance ratios trended lower in patients with metabolic syndrome (mean difference: − 4.4%, 95% CrI: − 11.0 to 2.3, posterior probability of decrease 90.3%) while there was no meaningful change in high V/Q impedance ratios. Pathway decomposition analysis estimated that approximately 85% of the predicted association of metabolic syndrome to low V/Q impedance ratios in dorsal lung regions was attributable to ventilation changes, with perfusion distribution accounting for 15%.

### Association between regional ventilation and perfusion distributions

**Fig. 3:**
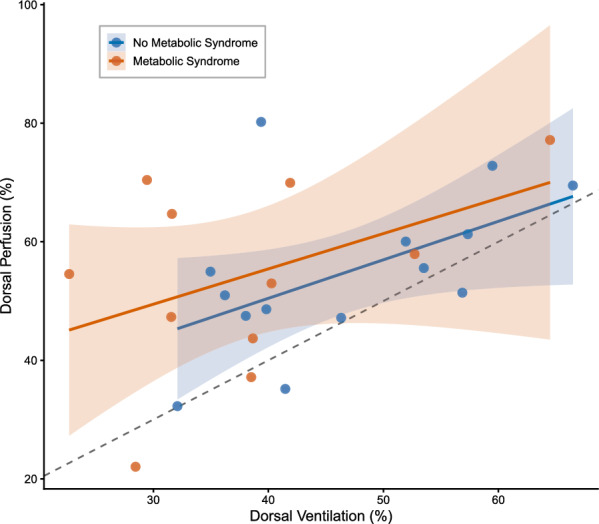
Association Between Regional Ventilation and Perfusion Distributions in Dorsal Lung Regions. Association between ventilation and perfusion distributions (% of total impedance) in dorsal lung regions across patients stratified by metabolic syndrome (red) and no metabolic syndrome (blue). The dashed line represents an equal ventilation and perfusion distribution % (slope = 1). Each point represents one patient; shaded regions indicate 95% confidence intervals for group-specific regression lines

The association between regional ventilation and perfusion distribution between patients is shown in Fig. 3. It did not differ by metabolic syndrome (interaction coefficient: 0.01, 95% CrI: − 0.34 to 0.36). Regression slopes were nearly identical in both groups (1.07 vs 1.06), indicating that across patients, perfusion distribution scaled proportionally with ventilation distribution regardless of metabolic syndrome.

### Sensitivity analyses

Additional adjustment for sex did not change the results (effect estimates changed < 10%). When BMI was added to the model, the association between metabolic syndrome and ventilation distribution to dorsal lung regions was partially attenuated (BMI-adjusted difference: − 3.48%, 95% CrI: − 10.98 to 4.06), with approximately 40% of the total effect mediated by BMI.

## Discussion

In a retrospective single-center cohort study of 25 patients with ARDS, we characterized regional ventilation and perfusion distributions stratified by metabolic syndrome using electrical impedance tomography. Compared to patients without metabolic syndrome, patients with metabolic syndrome had reduced ventilation distribution with smaller changes in perfusion distribution in dorsal lung regions. The association between regional ventilation and perfusion distributions was similar between groups. These findings raise the hypothesis that metabolic syndrome may alter regional lung physiology in ARDS primarily through mechanisms that affect ventilation distribution.

In this exploratory analysis, metabolic syndrome was associated with reduced ventilation distribution in dorsal lung regions. This pattern is consistent with central adiposity increasing thoracic mass loading and promoting dependent lung collapse [30]. Our definition of central obesity used BMI ≥ 35 kg/m^2^ as a surrogate for waist circumference, which is not routinely measured in critically ill patients, creating substantial overlap between metabolic syndrome classification and severe obesity. Our sensitivity analysis estimated that BMI accounted for approximately 40% of the association between metabolic syndrome and reduced dorsal ventilation, suggesting that mechanical loading from adiposity is an important but not sole contributor. The remaining association may reflect non-mechanical components of metabolic syndrome, including systemic inflammation and endothelial dysfunction, that contribute to alveolar edema and impaired lung recruitment. However, the small sample size limits the robustness of this decomposition, and future studies with larger cohorts could compare obesity-based versus metabolic syndrome-based stratifications to better isolate these distinct pathways. Dorsal ventilation distribution remained reduced in metabolic syndrome despite recruitment maneuvers and decremental PEEP titration aimed at optimizing respiratory system compliance. Our results are consistent with a porcine model of lung injury demonstrating that external chest and abdominal loading impairs the ability of mechanical ventilation to restore lung mechanics to unloaded conditions in the supine position [31]. Notably, patients with metabolic syndrome required higher PEEP after titration (14.9 vs 11.1 cmH₂O), consistent with the need for greater pressure to counteract increased chest wall and abdominal loading. Despite higher optimized PEEP and lower residual collapse on EIT, the persistent ventral shift in ventilation distribution suggests that PEEP titration alone may incompletely overcome the mechanical disadvantage imposed by central adiposity in the supine position.

In contrast to ventilation, perfusion distribution showed modest and uncertain differences between groups in dorsal lung regions. The smaller change in perfusion, combined with reduced ventilation, resulted in a trend toward an increase in low V/Q impedance ratios in the dorsal lung. Further analysis clarified the relative contributions of ventilation and perfusion changes where approximately 85% of the predicted change was attributable to reduced ventilation, with perfusion redistribution accounting for 15%. The reduction in dorsal ventilation in combination with the absence of meaningful changes in regional perfusion suggests that physiologic ventilation-to-perfusion mismatch in patients with metabolic syndrome may be primarily driven by impaired ventilation rather than perfusion redistribution. Notably, the association between ventilation and perfusion distribution did not differ by metabolic syndrome. Perfusion distribution decreased proportionally with ventilation distribution in both groups. Our data suggest that hypoxemia in this population likely arises predominantly from persistent reductions in dorsal ventilation, while lung perfusion remains relatively preserved.

Our study has several strengths. First, the integration of EIT into routine clinical practice at our institution enables bedside, radiation-free assessment of regional ventilation and perfusion [15, 20]. Second, our focus on patients with metabolic syndrome addresses an understudied subgroup of ARDS patients with increased vulnerability to respiratory failure. Third, we optimized mechanical ventilation using a recruitment maneuver and decremental PEEP trial prior to EIT acquisition, reducing the influence of suboptimal ventilatory management on regional distributions. Prior evidence indicates that this approach improves lung mechanics in patients with obesity [13]. Finally, our use of Bayesian statistical methods allowed estimation of effects and uncertainty despite modest sample size.

Our study has several limitations. First, our single-center design limits generalizability. The Lung Rescue Team at the Massachusetts General Hospital does not evaluate all patients with ARDS, which introduces selection bias. Second, residual confounding is likely in an observational study design. Patients with metabolic syndrome were younger than those without metabolic syndrome. We adjusted for age in our primary analyses to account for this potential confounder. Third, EIT measures relative rather than absolute perfusion as we did not have synchronous cardiac output data and cannot quantify true physiologic shunt and dead space. However, prior work from our group has validated its ability to track regional perfusion changes over time and in response to inhaled nitric oxide, supporting its use here [13, 14]. Nevertheless, EIT estimates are susceptible to motion artifact, do not account for lung density (e.g., dependent lung compression) that influences measures of regional perfusion [32]. Fourth, we did not capture more specific measures of adiposity and metabolic dysfunction such as waist circumference and hemoglobin A1c levels that may have stronger correlations to vascular impairment. Fifth, our analysis of the association between regional ventilation and perfusion distributions reflects between-patient associations and does not directly assess dynamic perfusion responses to hypoxemia or interventions. Lastly, given the small sample size and posterior uncertainty around several estimates, all findings should be considered hypothesis-generating and require confirmation in larger cohorts.

In conclusion, metabolic syndrome may be associated with reduced ventilation distribution to dorsal lung regions in ARDS with smaller changes in perfusion. The association between regional ventilation and perfusion distribution did not differ by metabolic syndrome. These findings suggest distinct regional physiologic abnormalities may exist in patients with metabolic syndrome, a population at high risk for ARDS development and severe disease. Prospective studies with biomarker and outcome assessment are needed to establish mechanistic pathways and clinical relevance.

## Supplementary Information

Additional file1 (DOCX 33 KB)

Additional file2 (DOCX 15 KB)

Additional file3 (PDF 1104 KB)

Additional file4 (JPEG 113 KB)

## Data Availability

Deidentified datasets, EIT imaging, and analytic code are available upon reasonable request to the corresponding author, subject to data use agreement and evaluation by the Respiratory Care and Lung Rescue Directors at the Massachusetts General Hospital.

## Abbreviations

ARDS: Acute Respiratory Distress Syndrome
EIT: Electrical Impedance Tomography
PEEP: Positive End-Expiratory Pressure
FiO₂: Fraction of Inspired Oxygen
PaO₂: Partial Pressure of Oxygen in Arterial Blood
HDL: High-Density Lipoprotein
APACHE: Acute Physiology and Chronic Health Evaluation
SMD: Standardized Mean Difference
SD: Standard Deviation
IQR: Interquartile Range
CrI: Credible Interval
BMI: Body Mass Index
V/Q: Ventilation to Perfusion
