# Supplementary material for: The impact of metabolic syndrome on regional ventilation and perfusion in ARDS: an observational cohort study using electrical impedance tomography
Source: Intensive Care Med Exp. 2026 Mar 11;14:34. doi: 10.1186/s40635-026-00883-8 (PMC12979738; doi:10.1186/s40635-026-00883-8)
Supplement: Supplementary file 2 — Additional file2 (DOCX 15 KB) [file 40635_2026_883_MOESM2_ESM.docx]

We specified weakly informative priors prior to data analysis based on physiological constraints and prior EIT literature in ARDS, following recommendations for Bayesian analysis in critical care medicine. (Goligher 2024) For regional ventilation and perfusion distribution, we used normal(50, 30) intercept priors, reflecting that ventilation and perfusion distribute approximately equally between dorsal and ventral regions in healthy subjects, with prior studies in ARDS reporting dorsal ventilation ranging from 35-55% depending on disease severity and PEEP settings. (Spinelli 2021) This prior assigns 95% probability to values between -10% and 110%, encompassing the full range of observed dorsal ventilation values in ARDS (35-55%) while allowing flexibility beyond empirical bounds. For V/Q impedance ratios, we used normal(25, 25) intercept priors based on reported values of 10-40% for shunt and dead space fractions in ARDS patients. (Spinelli 2021, Leali 2024)

For regression coefficients including the metabolic syndrome effect, we specified normal(0, 5) priors centered on no effect. These priors assign 95% probability to effects between -10% and +10%, which spans clinically meaningful differences in regional distribution. The equivalent prior sample size is approximately 9 observations (calculated as σ²_data / σ²_prior ≈ 15² / 5² = 9), indicating that even modest data will dominate inference. (Goligher 2024)

For residual standard deviations, we used exponential(0.1) priors for Gaussian models and exponential(0.05) for Student-t models. These priors have means of 10% and 20% respectively, consistent with observed inter-patient variability in EIT-derived regional distributions. (Spinelli 2021) For Student-t degrees of freedom (ν), we specified gamma(4, 1), yielding a mean of 4 with 95% probability between 1 and 10, allowing for heavy-tailed distributions when indicated by the data.

Prior predictive checks confirmed that these priors generated physiologically plausible outcome distributions before observing data.

Spinelli E, Kircher M, Stender B, et al., (2021) Unmatched ventilation and perfusion measured by electrical impedance tomography predicts the outcome of ARDS. Crit Care 25: 192. PMC8173510. PMID: 34082795. PMCID: PMC8173510. DOI: 10.1186/s13054-021-03615-4.

Goligher EC, Harhay MO, (2024) What Is the Point of Bayesian Analysis? Am J Respir Crit Care Med 209: 485-487. PMC10919113. PMID: 37922491. PMCID: PMC10919113. DOI: 10.1164/rccm.202310-1757VP.

Leali M, Marongiu I, Spinelli E, et al., (2024) Absolute values of regional ventilation-perfusion mismatch in patients with ARDS monitored by electrical impedance tomography and the role of dead space and shunt compensation. Crit Care 28: 241. PMC11251389. PMID: 39010228. PMCID: PMC11251389. DOI: 10.1186/s13054-024-05033-8.
