# Supplementary figures and images for: The impact of metabolic syndrome on regional ventilation and perfusion in ARDS: an observational cohort study using electrical impedance tomography
Source: Intensive Care Med Exp. 2026 Mar 11;14:34. doi: 10.1186/s40635-026-00883-8 (PMC12979738; doi:10.1186/s40635-026-00883-8)

Observed data      Posterior predictions (n = 50)

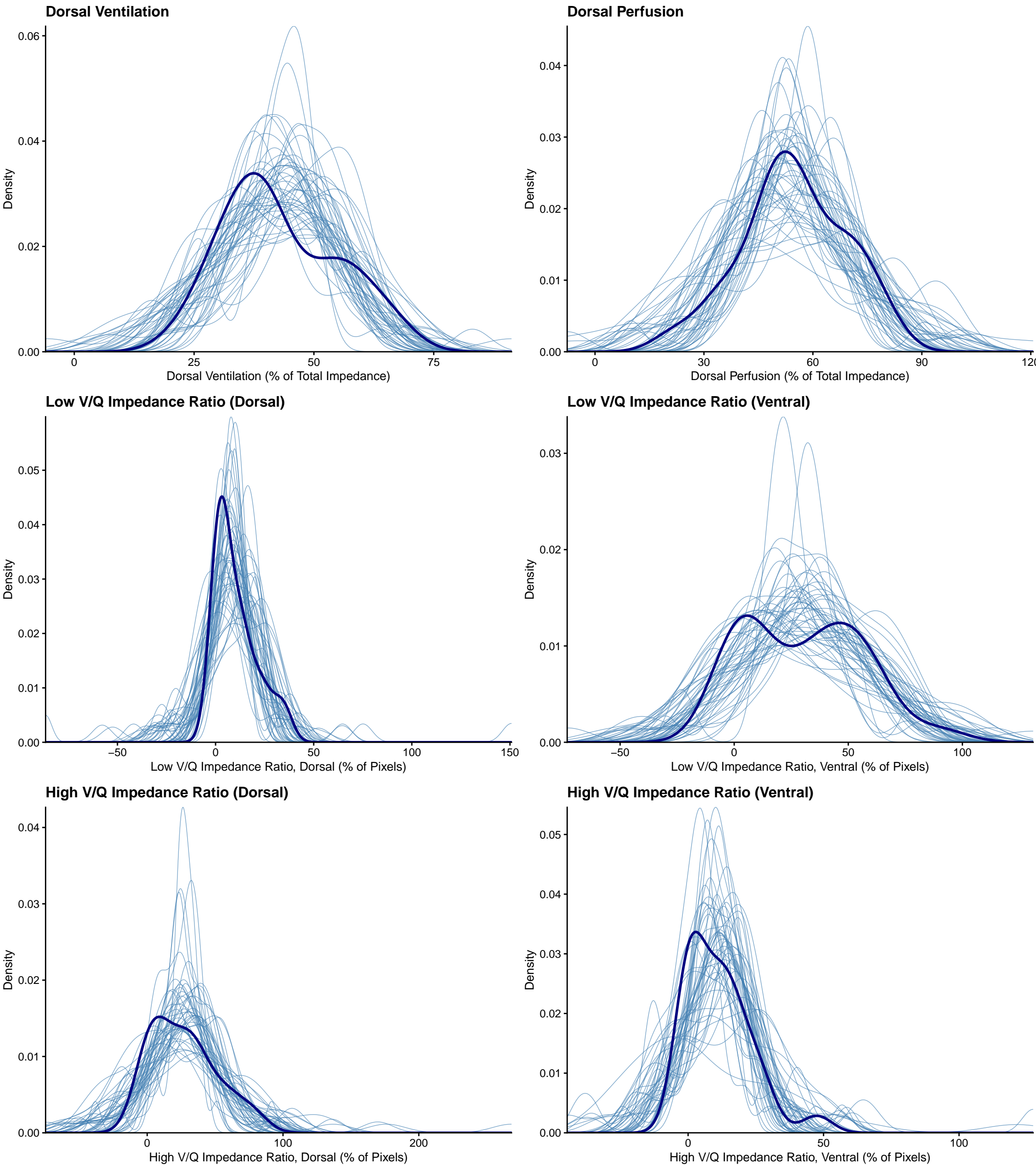

Supplement: Supplementary file 3 — Additional file3 (PDF 1104 KB) [file 40635_2026_883_MOESM3_ESM.pdf]

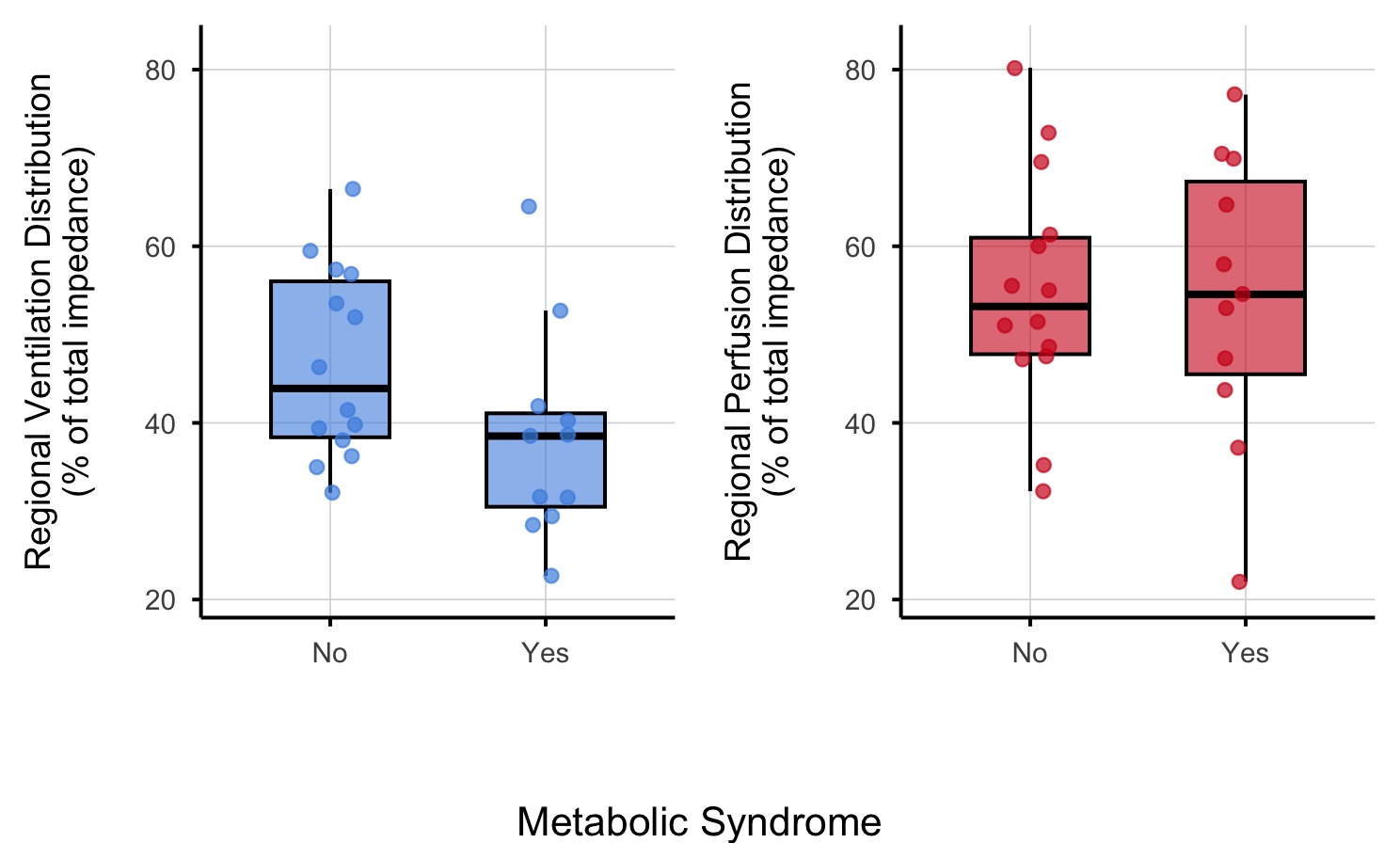

Supplement: Supplementary file 4 — Additional file4 (JPEG 113 KB) [file 40635_2026_883_MOESM4_ESM.jpeg]
